# Preparation and thermal conductivity of CuO nanofluid via a wet chemical method

**DOI:** 10.1186/1556-276X-6-181

**Published:** 2011-02-28

**Authors:** Haitao Zhu, Dongxiao Han, Zhaoguo Meng, Daxiong Wu, Canying Zhang

**Affiliations:** 1 College of Materials Science & Engineering, Qingdao University of Science & Technology, Qingdao, 266042, China

## Abstract

In this article, a wet chemical method was developed to prepare stable CuO nanofluids. The influences of synthesis parameters, such as kinds and amounts of copper salts, reaction time, were studied. The thermal conductivities of CuO nanofluids were also investigated. The results showed that different copper salts resulted in different particle morphology. The concentration of copper acetate and reaction time affected the size and shape of clusters of primary nanoparticles. Nanofluids with different microstructures could be obtained by changing the synthesis parameters. The thermal conductivities of CuO nanofluids increased with the increase of particle loading.

## Introduction

Nanofluid is a new class of heat transfer fluids containing nano-sized particles, fibers, or tubes that are stably suspended in a carrier liquid [1-4]. Since the concept of nanofluid was proposed [1], more and more researchers have been committing to it because of the thermal properties and the potential applications associated with heat transfer, mass transfer, wetting, and spreading [1-7].

Preparation of stable nanofluids is the first step and key issue of nanofluid research and applications. At present, some methods, such as dispersion method, direct evaporation condensation method (DECM), submerged-arc nanoparticles synthesis system (SANSS), laser ablation method, and wet chemical method, etc. [2-4,8-12], have been applied to synthesize nanofluids. Dispersion method is a two-step method [13-18], in which commercial nanoparticles are dispersed into base fluid under ultrasonic agitation or mechanical stirring. The advantage of this method is that it could prepare nanofluids in a large scale. However, nanoparticle aggregations are difficult to breakup under ultrasonication or stirring. Thus, stability and thermal conductivity of nanofluids prepared with dispersion method are usually not ideal. DECM, SANSS, and laser ablation method are one-step physical methods [19-22], in which metal materials are vaporized by physical technology and cooled into liquids to obtain nanofluids. These physical methods provide excellent control on the particle size and can produce stable nanofluids. However, it is difficult to synthesize nanofluids in a large scale. Our team has developed a wet chemical method with which several kinds of nanofluids have been produced successfully [23-25]. It has the advantages in terms of controlling the particle size, reducing agglomeration of the nanoparticles, and producing nanofluids in a large scale. This method is a promising technique for commercial synthesis of nanofluids. However, the research about the influences of synthesis parameters on nanofluids microstructure and properties are scarce, though it is very important for industrial synthesis of nanofluids.

In this study, CuO nanofluid was synthesized with a wet chemical method. The influences of synthesis parameters, such as kinds and amounts of copper salts, reaction time, were studied by X-ray diffraction (XRD), transmission electron microscopy (TEM), and particle size analyzer. The thermal conductivity of CuO nanofluids was also studied.

## Experimental section

**Figure 1:**
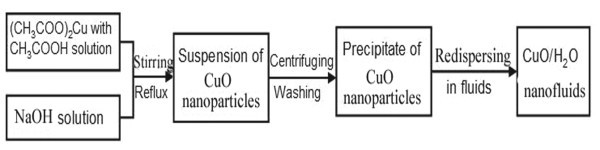
**Preparation process of CuO aqueous nanofluids**.

All of the reagents used in the experiment were of analytic purity. Figure 1 shows the preparation process. The synthesis process is based on the following chemical reactions in solution:

(1) $${\text{Cu}}^{2+}+2\text{NaOH}=\text{Cu}{\left(\text{OH}\right)}_{2}+2{\text{Na}}^{+}$$

(2) $$\text{Cu}{\left(\text{OH}\right)}_{2}≜\text{CuO}+{\text{H}}_{2}\text{O}$$

In a typical procedure, 600 ml 0.2 M copper acetate (Cu(CH_3_COO)_2_·H_2_O) solution and 2 ml glacial acetic acid (CH_3_COOH) were added into a round-bottomed flask and heated to boiling under magnetic stirring. Then, 30 ml 8 M sodium hydroxide (NaOH) solution was poured into the flask. The color of the solution turned from blue to black immediately, and a black suspension formed simultaneously. The reaction was carried out under stirring and boiling for 2 h. The mixture was cooled to room temperature and centrifuged. Then, a wet CuO precipitate was obtained. The wet precipitate was washed twice with distilled water to remove the impurity ions. CuO nanofluids of different volume fractions were obtained by re-dispersing the wet precipitate into different amounts of distilled water under ultrasonic vibration (120 W, 40 Hz).

To study the influences of synthesis parameters on the final products, the kinds and amounts of copper salts, reaction time were changed while keeping all other experimental parameters same as in the typical run.

The XRD pattern of the powder (obtained by drying the washed wet precipitate) was recorded on a Rigaku D/Max r-A diffractometer. TEM images were captured on a JEM-2000EX instrument. The nanofluids were diluted with distilled water and dispersed by ultrasonic. Then, one drop was placed on a carbon-coated copper grid and left to dry at room temperature. Particle size distributions of the nanoparticles in nanofluids were measured with a Zetasizer 3000HS (Malvern) particle size analyzer. The samples were also prepared by diluting the nanofluids with distilled water and dispersed by ultrasonic. Thermal conductivity was measured using a KD2 Pro Thermal Property Analyzer (Decagon Inc., Pullman, WA, USA) based on the transient hot wire method. The nanofluids were sonicated for about 30 min before measurements so that the samples would have the same dispersity.

## Results and discussion

### Characterization of typical sample

**Figure 2:**
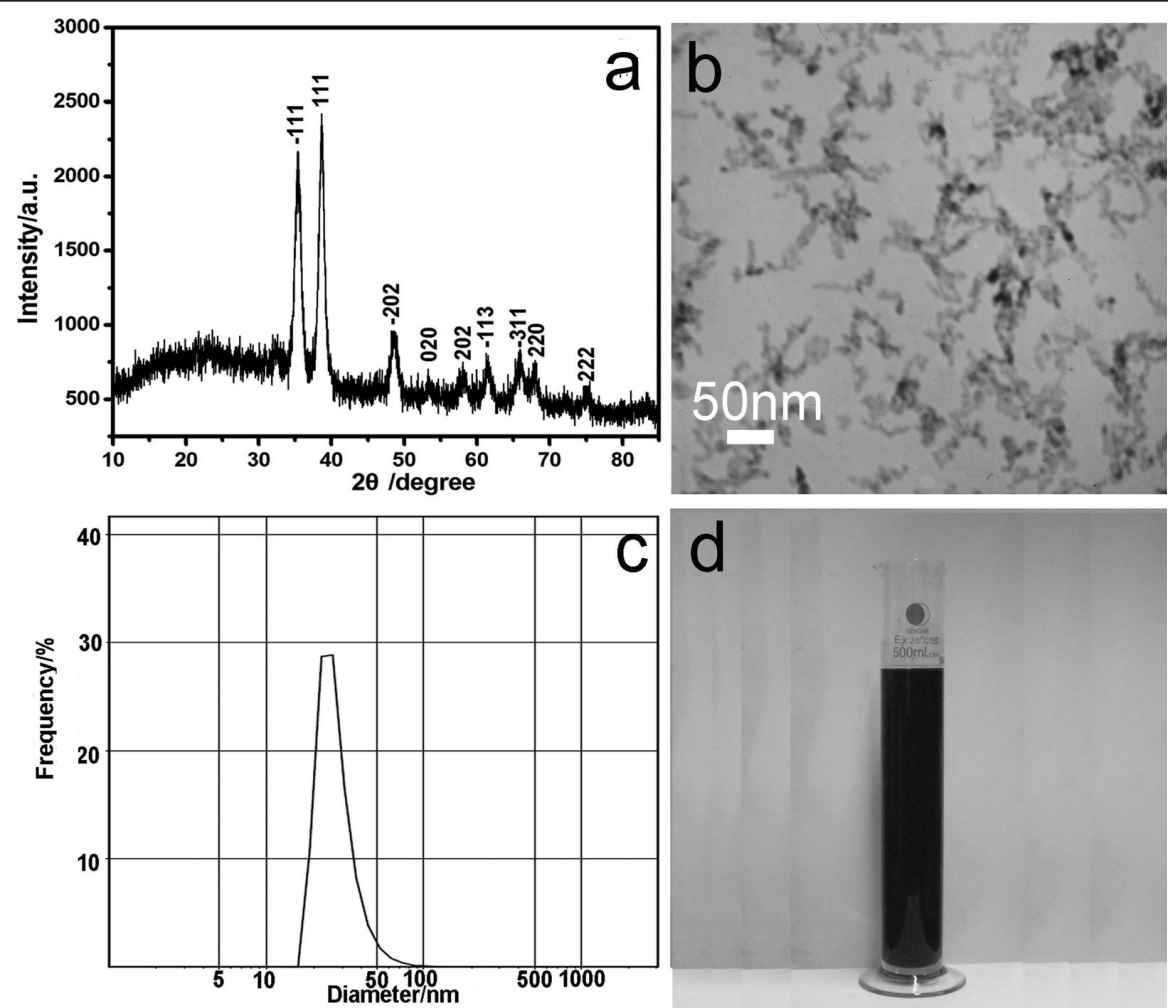
**Characterization of the typical sample**. **(a) **XRD pattern; **(b) **TEM image; **(c) **size distribution; **(d) **real photo.

Figure 2a is the XRD pattern of the typical sample. All the peaks on the XRD pattern can be indexed to that of monoclinic CuO according to the literature (JCPDS, FileNo 80-1916). The average crystal size is 10.4 nm calculated using Debye-Scherrer formula. Figure 2b shows a TEM image of the typical sample. The size of primary particles is about 10 nm, which is in good agreement with the result of XRD. The primary particles aggregate to chain-like clusters with width of 10 nm and length of 50-150 nm (5-15 primary particles). Figure 2c is the size distribution of the typical sample. The particle size is about 20-80 nm, and the size distribution is narrow. The larger particle size is due to the short clusters shown in the TEM image. Figure 2d is the real photo of the products. The obtained CuO nanofluids could remain stable for 5 months with no visible precipitation at the bottom.

### Influences of copper salts

**Figure 3:**
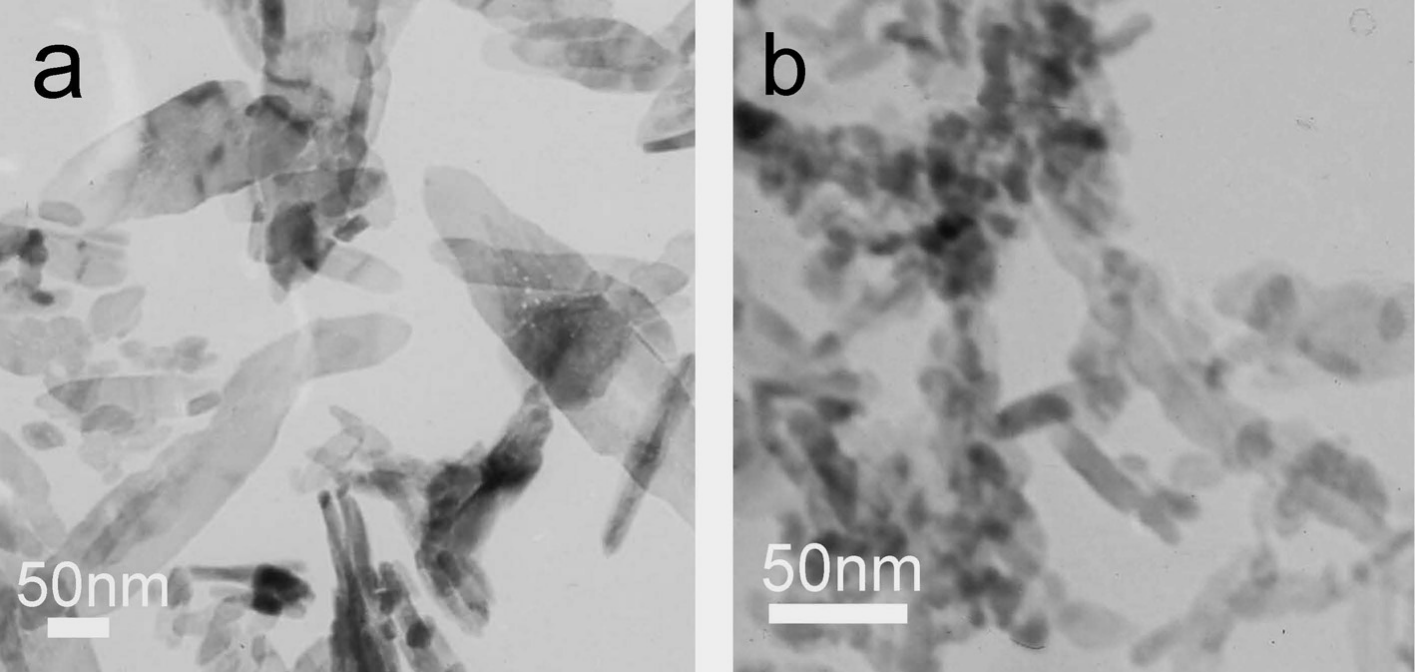
**TEM images of CuO nanofluids prepared with different copper salts**. **(a) **CuCl_2_·2H_2_O; **(b) **Cu(NO_3_)_2_·3H_2_O.

By replacing Cu(CH_3_COO)_2_·H_2_O with CuCl_2_·2H_2_O and Cu(NO_3_)_3_·3H_2_O, respectively, different CuO nanofluids were prepared with all other experimental parameters unchanged. Figure 3 is the TEM images of above two nanofluids. When using CuCl_2_·2H_2_O as copper source (Figure 3a), the obtained particles in nanofluids are flake-like particles with width of 10-80 nm and length of 100-300 nm. When using Cu(NO_3_)_2_·3H_2_O (Figure 3b), the particles are aggregations of thin sticks and particles of about 15-50 nm. It has been approved by some researchers that the anions could affect the growth orientation and process of nanoparticles by adsorption or coordination interaction of anions with special crystal face of particles [26]. Therefore, by changing copper source, we could obtain particles with different morphology.

### Influences of copper acetate concentration

**Figure 4:**
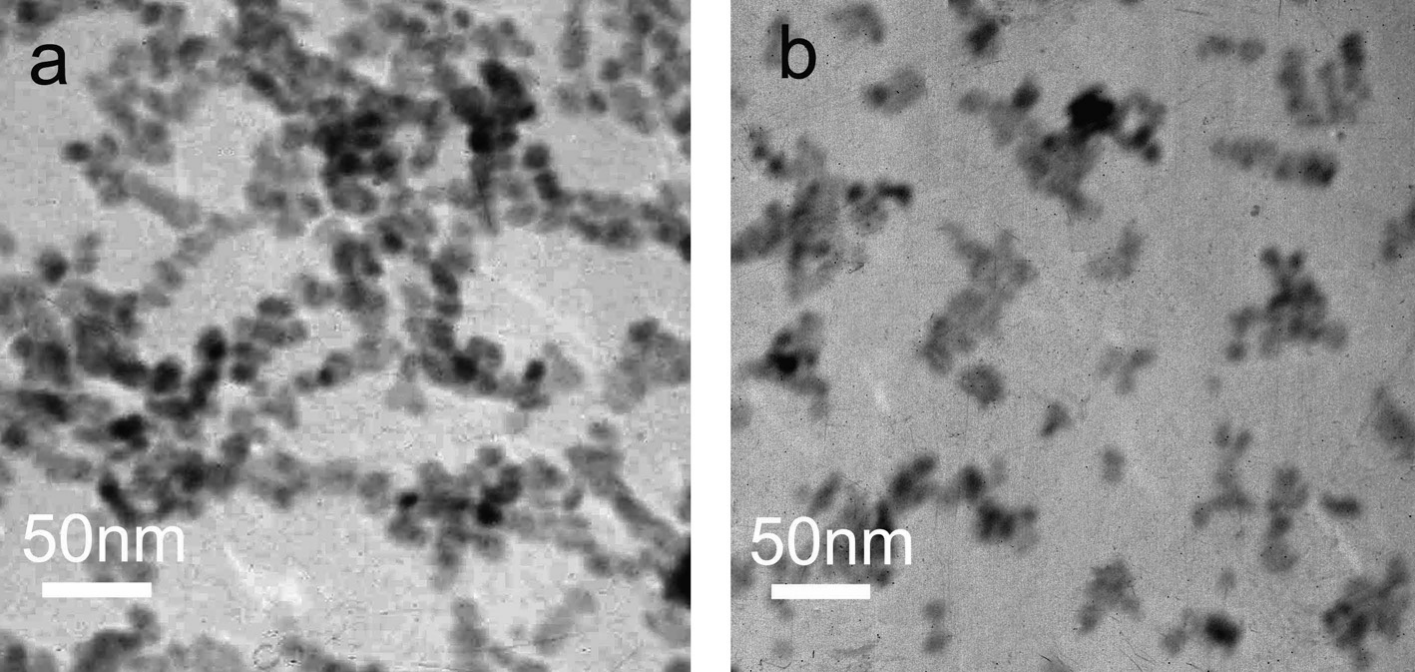
**TEM images of CuO nanofluids prepared with different concentrations of (CH**_**3**_**COO)**_**2**_**Cu·H**_**2**_**O solution**. **(a) **0.1 mol·l^-1^; **(b) **0.4 mol·l^-1^.

Figure 4a,b are the TEM images of CuO nanofluids prepared with copper acetate concentration of 0.1 and 0.4 mol·l^-1^, respectively. Compared with typical nanofluids (obtained with concentration of 0.2 mol·l^-1^), it is clear that the size of primary nanoparticles remain almost the same (about 10 nm), but the morphology and size of nanoparticles cluster change with copper acetate concentration. When the concentration is 0.1 mol·l^-1^, the clusters are also chain-like structures with lengths in the range of 100-200 nm. It is longer than the clusters in typical samples. When the concentration is 0.4 mol·l^-1^, the primary nanoparticles aggregate and form irregular clusters consisted of 2-30 primary nanoparticles. The formation of chain-like cluster may be due to the orientation adhesion mechanism [27]. When the concentration of copper acetate is low, the collision probability of primary CuO nanoparticles is low; thus, the orientation adhesion is preponderant in the reaction process. Therefore, by changing the concentration of copper acetate, the size and structure of cluster could be adjusted.

### Influence of reaction time

**Figure 5:**
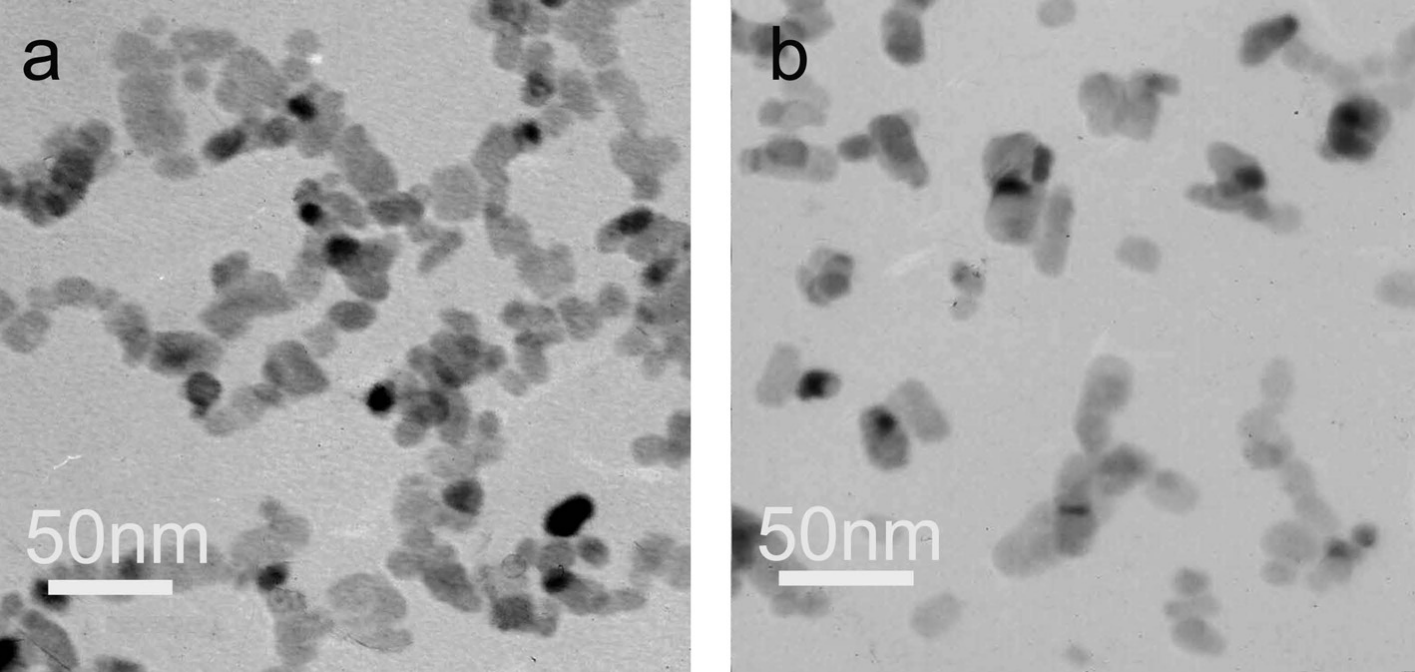
**TEM images of CuO nanofluids synthesized under different reaction times**. **(a) **12 h; **(b) **25 h.

Figure 5 is the TEM images of CuO nanofluids obtained with different reaction times. When the reaction time is 12 h (Figure 5a), average size of CuO primary nanoparticles is about 10 nm. CuO nanoparticles form flexural chains consisting of 30-50 primary particles. It is longer than the chain in typical sample (Figure 2b). When the reaction time was increased to 25 h (Figure 5b), the size of the primary particles is also about 10 nm, but the chain-like clusters do not exist any more. Instead, there are small aggregates composed of several primary particles. As mentioned above, the formation mechanism of chain-like cluster is orientation adhesion. With the increase of reaction time, the orientation adhesion degree increases; and thus, the length of the cluster increases. Why do the chain-like clusters destroy when the reaction time is 25 h? It needs more detailed research in future studies. The above results show that different microstructures could be obtained through changing the reaction time.

### Thermal conductivity of CuO nanofluids

**Figure 6:**
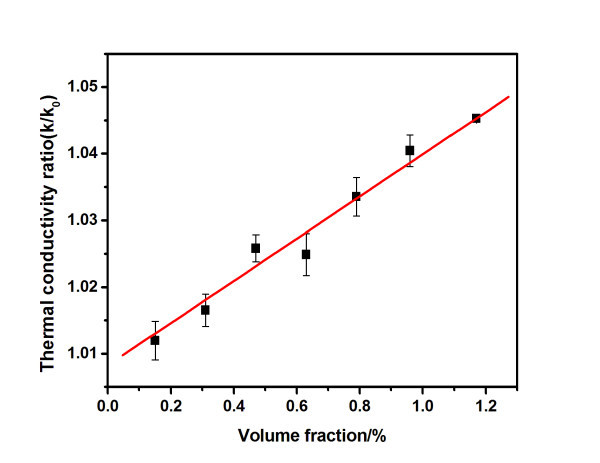
**Thermal conductivity ratio as a function of CuO volume fraction at 25°C**.

Figure 6 shows the thermal conductivity ratio of the typical sample, defined as *k*/*k*_0_, where *k *and *k*_0 _are the thermal conductivities of the nanofluids and the base media (H_2_O) respectively, as a function of the particle volume fraction at 25°C. The thermal conductivity of the base fluid (H_2_O) was measured, and it had an average value of 0.580 W·m^-1^·K^-1^. It can be seen that the thermal conductivity ratio increases as the particle volume fraction increases. This is in good agreement with some research, in which the thermal conductivity of nanofluids also increase linearly with the particle loading [28,29]. On comparing with some reported experimental results of CuO nanofluids, the current data are found to be close to Lee et al.'s data, Das et al.'s data, and Liu et al.'s data [30-32], suggesting the potential application as heat transfer fluids.

## Conclusion

A wet chemical method to synthesize stable CuO nanofluids in a large-scale was developed successfully. The influences of synthesis parameters on nanofluids microstructures were investigated. Different copper salts resulted in different particle morphologies. The concentration of copper acetate and reaction time affected the size and shape of clusters of primary nanoparticles. Nanofluids with different microstructures could be obtained through changing the synthesis parameters. The thermal conductivity of CuO nanofluids increased with the increase of particle loading. It is expected that this method can be extended to synthesize other nanofluids.

## Abbreviations

DECM: direct evaporation condensation method; SANSS: submerged arc nanoparticles synthesis system; TEM: transmission electron microscopy; XRD: X-ray diffraction.

## Competing interests

The authors declare that they have no competing interests.

## Authors' contributions

HZ designed and guided all aspects of the work. DH carried out the experiments and drafted the manuscript. ZM, DW and CZ participated in the design of the study and revised the manuscript.
